# Interactions Between *Arma chinensis* and Entomopathogenic Nematodes for Biological Control of *Tuta absoluta*

**DOI:** 10.3390/insects17060627

**Published:** 2026-06-14

**Authors:** Yan Zhao, Maiqi Shi, Yuyang Jiang, Qian Chen, Ruize Li, Wen Meng, Youming Hou, Sheng-Yen Wu

**Affiliations:** State Key Laboratory of Agricultural and Forestry Biosecurity, Fujian Agriculture and Forestry University, Fuzhou 350002, China; zhaoyan1996@126.com (Y.Z.);

**Keywords:** tomato leafminer, predatory bug, EPNs, non-target effect, additive interaction

## Abstract

The tomato leafminer *Tuta absoluta* (Meyrick) is a devastating invasive pest that threatens tomato production worldwide. Reliance on chemical insecticides raises sustainability concerns, highlighting the need for effective biological alternatives. Combining predators with entomopathogenic nematodes (EPNs) represents a promising strategy, yet their interactions remain poorly characterized. Here, we conducted laboratory bioassays to assess the individual and joint effects of the predatory bug *Arma chinensis* (Fallou) and four EPN species, *Steinernema carpocapsae*, *S. feltiae*, *S. riobrave*, and *Heterorhabditis bacteriophora*, against *T. absoluta* larvae. Under these controlled conditions, *H. bacteriophora* showed the highest compatibility with *A. chinensis*, exhibiting the lowest virulence against the predator. Female *A. chinensis* exhibited strong predation on freely exposed second-instar larvae, but efficiency declined markedly against leaf-mining larvae. *Heterorhabditis bacteriophora* caused consistently high mortality in second instars regardless of protection. Their combined application resulted in additive mortality with significantly reduced LT_50_ values. We also observed *A. chinensis* preying on nematode-infected larvae and occasional infection of the predator under confined conditions. These laboratory findings demonstrate additive effects against *T. absoluta*, providing preliminary evidence for stage-specific integrated biological control strategies.

## Simple Summary

The tomato leafminer *Tuta absoluta* is a devastating pest that affects tomato production worldwide. Reliance on chemical insecticides raises sustainability concerns, highlighting the need for biological alternatives. In this laboratory study, we assessed the combined application of the predatory bug *Arma chinensis* and entomopathogenic nematodes against *T. absoluta* larvae. *Heterorhabditis bacteriophora* showed the lowest virulence against the predator among the tested nematodes. Female *A. chinensis* exhibited strong predation on exposed larvae, but efficiency declined against leaf-mining larvae. *Heterorhabditis bacteriophora* caused high mortality in early instars regardless of leaf-mining protection. Their combined application produced additive effects with reduced LT_50_ values. We also observed *A. chinensis* preying on nematode-infected larvae. These findings demonstrate that combining these natural enemies can enhance pest control, providing a basis for integrated management strategies.

## 1. Introduction

The tomato leafminer, *Tuta absoluta* (Meyrick) (Lepidoptera: Gelechiidae), is a devastating invasive pest of tomato originating from South America and now established worldwide [1,2]. Its concealed larval feeding and high reproductive capacity can result in severe yield losses if unmanaged. Chemical insecticides, the mainstay of control, have led to resistance development and non-target impacts, undermining sustainability [3,4]. With rising demands to reduce pesticide use, interest has shifted toward ecologically sound alternatives [5]. Biological control agents, such as *Trichogramma* parasitoids, show promise, but their field performance is constrained by climatic factors and host specificity [6,7,8]. Thus, developing robust biological control strategies is a priority in integrated pest management (IPM) for *T. absoluta*.

Currently, biological control of *T. absoluta* relies mainly on mirid predators such as *Nesidiocoris tenuis* (Reuter) and *Macrolophus pygmaeus* (Rambur), which are effective against eggs and early instars but less so against later larval stages [9,10,11]. Their use in augmentative release programs can reduce crop damage, though establishment often requires supplemental feeding [12,13]. Beyond these hemipterans, the asopine predator *Arma chinensis* (Fallou) has demonstrated broad efficacy against lepidopteran pests such as *Spodoptera litura* (Fabricius) and *Plodia interpunctella* (Hübner), aided by its facultative use of plant resources [14,15]. However, its potential against *T. absoluta*—including predation across larval instars, ability to access leaf-mining larvae, and compatibility with other biocontrol agents—remains poorly understood.

Entomopathogenic nematodes (EPNs) of the genera *Steinernema* and *Heterorhabditis* are promising biological control agents applied via soil or foliar treatments [16,17]. These nematodes vector symbiotic bacteria (*Xenorhabdus* spp. or *Photorhabdus* spp.) that rapidly kill insect hosts within 24–48 h [18,19]. Recent studies have shown efficacy against *T. absoluta* [20,21], and highlighted advances in foliar application through protective formulations that enhance survival and target penetration [22,23]. However, broader field performance is often constrained by environmental sensitivity, particularly ultraviolet (UV) radiation and desiccation, which can rapidly reduce nematode survival and infectivity when applied to foliage, and limited penetration of leaf-mining stages [24,25,26].

Ecological interactions between EPNs and other natural enemies range from synergistic to antagonistic. Plant-derived compounds can affect nematode virulence [27], and predatory arthropods may reduce EPN populations by consuming infected cadavers [28]. Mutualistic interactions also exist, e.g., *Fusarium solani* (Martius) attracts EPNs and enhances their efficacy [29,30]. Aboveground, intraguild predation among mirids and parasitoids can influence biological control [31,32]. Crucially, EPNs can have direct non-target effects on beneficial predators like *N*. *tenuis* [33,34]. This highlights the importance of assessing compatibility before integrating EPNs with other biocontrol agents within an IPM framework. Yet, despite this evidence, empirical data on interactions between hemipteran predators and EPNs, particularly regarding *T. absoluta*, remain scarce.

Therefore, an evaluation of their basic compatibility and joint effects is a necessary first step. Based on this, we hypothesize that certain EPNs will exhibit low virulence to *A. chinensis*, thereby facilitating predator attack and yielding additive or synergistic suppression of *T. absoluta*. To test this, we conducted a series of laboratory bioassays to (i) evaluate potential non-target effects of four EPN species on *A. chinensis*, (ii) quantify stage-specific predation on leaf-mining and exposed larvae, and (iii) analyze mortality dynamics in combined treatments. Our study addresses critical gaps in the integration of predators and EPNs, aiming to advance sustainable IPM strategies against this invasive pest.

## 2. Materials and Methods

### 2.1. Insect Rearing and EPN Culture

The stock culture of *T. absoluta* was derived from field-collected larvae infesting tomato plants (May 2024 in Yunnan, China). The moths were reared on potted tomato seedlings (cv. “Zhefen”) in climate-controlled chambers (HWS-250, Ningbo, China) at 25 ± 0.5 °C, 65 ± 5% RH, with a photoperiod of 16L:8D (light:dark) [35]. Adult moths were maintained in mesh cages (40 × 40 × 60 cm) and provided with 10% honey solution as a food source. Eggs were deposited on tomato leaves, which were replaced weekly to prevent overfeeding damage.

The *A. chinensis* population was purchased from Henan Jiyuan Baiyun Industrial Co., Ltd. (Jiyuan, China). All developmental stages of the predatory bugs were maintained in controlled environment chambers at 25 ± 0.5 °C, with 65 ± 5% RH and a photoperiod of 16L:8D. *Arma chinensis* were fed on *Tenebrio molitor* (Linnaeus) pupae. Experiments were conducted after two weeks of laboratory acclimatization.

Four EPN species, *S. carpocapsae* (All strain, Sc), *S. feltiae* (SN strain, Sf), *S. riobrave* (7–12 strain, Sr), and *H. bacteriophora* (H06 strain, Hb), were used in this study. The original populations of these nematodes were provided by the Research Center for Resource Insects and Bioengineering, Institute of Zoology, Guangdong Academy of Sciences, and the College of Life Sciences, Nankai University. These species have been maintained and propagated in laboratory culture for multiple years with confirmed taxonomic identities. All strains were subsequently cultured and maintained at the State Key Laboratory of Agricultural and Forestry Biosecurity, Fujian Agriculture and Forestry University. They were cultured and maintained using waxworm, *Galleria mellonella* (Linnaeus), larvae in 24-well plates. Plates were incubated at 25 °C until larval death (2–3 days) [36], after which cadavers were transferred to White traps [37]. Emerging IJs were collected after 8–9 days at 25 °C. The IJs were collected in 50 mL cell culture flasks, and stored in sterile distilled water at 15 °C in complete darkness for up to two weeks. Before each experiment, IJ activity was confirmed under a stereomicroscope (Nikon ECLIPSE Ts2, Tokyo, Japan).

### 2.2. Effects of EPNs on the Non-Target Predator A. chinensis

To assess the inherent virulence of EPNs on the non-target predator *A. chinensis*, a bioassay was conducted in a standardized, controlled laboratory system. The assay was conducted using 24-well plates containing 5 g of sterile quartz sand substrate (10% moisture) per well to simulate natural soil conditions. Nematode suspensions containing 25, 50, 100, or 200 IJs per well of each species were injected into the sand. One bug (second-instar, fifth-instar nymph, or adult) was placed in each well, with ten individuals per treatment. Control groups received 50 μL of distilled water. Mortality was recorded daily for 10 days. During this period, *A. chinensis* was provided with unlimited *T*. *molitor* pupae as food. Nematode infection was confirmed by transferring dead bugs to White traps and incubating at 25 °C for emergence observation. The experiment included five replicates and was independently conducted twice. Additionally, mortality of *A. chinensis* was recorded in control treatments without EPNs.

### 2.3. Predation Efficiency of A. chinensis on T. absoluta Larvae

**Figure A1:**
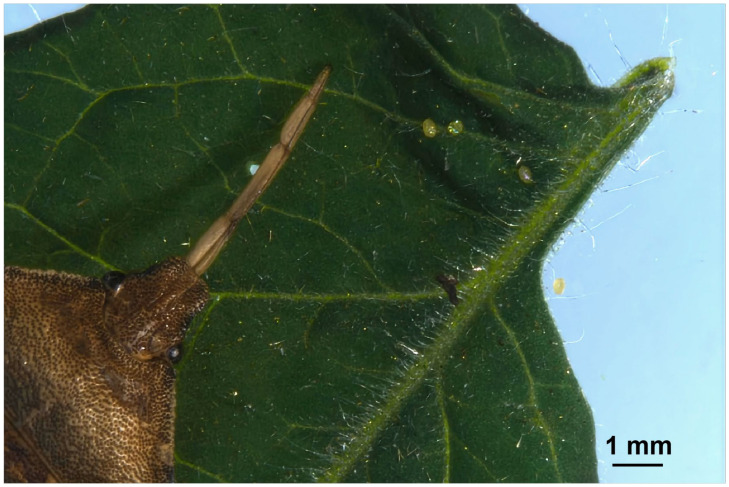
Morphological comparison between the stylet of the predatory bug *Arma chinensis* and the eggs of the tomato leafminer *Tuta absoluta*.

Following preliminary experiments, we excluded combinations involving *A. chinensis* with eggs of *T. absoluta* due to size mismatch (see Figure A1 for morphological comparison), as well as combinations with adult *T. absoluta* due to their ability to fly, which rendered them unsuitable prey. Additionally, we excluded early *A. chinensis* nymphs as predators, since they demonstrated low predation efficiency in our preliminary trials, corroborating the findings of Yang et al. (2023) on the performance of the predatory stink bug *Picromerus lewisi* (Scott) against *T. absoluta* [38]. Therefore, our predation bioassays focused on evaluating the intrinsic predatory capacity of actively foraging *A. chinensis* (fifth-instar nymphs and both female and male adults) against the key damaging larval stages of *T. absoluta*, including both early (second instar) and late (fourth instar) developmental phases. Furthermore, considering the leaf-mining behavior of *T. absoluta*, we additionally investigated predation on larvae that were either freely exposed on moist filter paper or sheltered within mined tomato leaf discs. Predation bioassays were conducted under laboratory conditions in 9 cm Petri dishes, each containing 15 *T. absoluta* larvae. Non-leaf-mining larvae were placed on moist filter paper, whereas leaf-mining larvae were provided with tomato leaf discs. After starving for 24 h, a single predator was introduced into each dish. Predation by *A. chinensis* was recorded 24 h later. The number of prey consumed was determined by counting the dead larvae that had been completely emptied of body contents. Each treatment was replicated five times in two independent trials. Control treatments without predators were included to account for natural mortality.

### 2.4. Effect of EPNs on T. absoluta Larvae

To assess the direct virulence of EPNs against *T. absoluta* larvae under standardized, no-choice conditions, a single *T. absoluta* larva was placed in each well of a 24-well plate and treated with 100 µL of distilled water containing 25 IJs of each EPN species. Three larval treatments were evaluated: second-instar non-mining larvae, fourth-instar non-mining larvae, and second-instar leaf-mining larvae. This design allowed for the comparison of virulence across larval stages (second vs. fourth instar) and the assessment of EPNs’ ability to infect larvae within the physical refuge of a leaf mine. For non-mining treatments, one target larva was transferred to each well along with a fresh tomato leaf fragment. For leaf-mining treatment, larvae were pre-established in tomato leaves for 24 h before assay initiation, after which the intact infested leaves containing one larva were transferred into each well. Control groups received 100 µL of sterile water without EPNs. For each treatment, 10 replicates in five trials were conducted across two independent experimental runs. Mortality was assessed at 12 h intervals for 72 h. Dead larvae displaying characteristic infection symptoms were transferred to individual White traps for nematode emergence confirmation.

### 2.5. Combined Effect of A. chinensis and EPNs on T. absoluta Larvae

Based on the non-target virulence assay (Section 2.2), *H. bacteriophora* was selected for combination trials as it showed the lowest virulence against *A. chinensis* and thus the highest compatibility. Combination treatments of *A. chinensis* adults and *H. bacteriophora* were conducted in a controlled, contained environment (500 mL sealed containers lined with filter paper) to assess their interactive effects on *T. absoluta* larvae. Ten second-instar larvae were pre-conditioned for 24 h on fresh tomato leaves to establish feeding sites prior to treatment application. Four experimental treatments were evaluated: (1) *A. chinensis* alone (one female adult), (2) *H. bacteriophora* alone (125 IJs), (3) combined *A. chinensis* and *H. bacteriophora* (one female adult + 125 IJs), and (4) distilled water control. Each treatment included five replicates, and all containers were maintained for 156 h before final mortality assessment. The entire experiment was independently conducted twice.

Additionally, a supplementary experiment was conducted to evaluate whether *A. chinensis* would prey on nematode-killed larvae, a potential interaction niche between the predator and the nematode. *Arma chinensis* adults were starved for 24 h and then individually exposed to one *T. absoluta* larva that had been killed by *H. bacteriophora* infection (24 h post-infection). Predation behavior was observed over a 24 h period in 16 replicates.

### 2.6. Statistical Analysis

The mortality rate was corrected using the formula of Abbott (1925) [39]: Corrected mortality = (Treatment mortality-Control mortality)/(1-Control mortality). All statistical analyses were performed with GraphPad Prism v7.0 (GraphPad Software, San Diego, CA, USA), and results with *p* < 0.05 were considered statistically significant. All data are presented as mean ± standard error (SE) from a minimum of five independent replicates.

For EPN virulence assays against *A. chinensis*, data were analyzed by two-way ANOVA with EPN species and concentration as main factors, followed by Tukey’s HSD test. For *H. bacteriophora* specifically, concentration-dependent mortality across developmental stages was assessed by fitting a four-parameter logistic regression model [log(inhibitor) vs. normalized response] to estimate median lethal concentrations (LC50) with 95% confidence intervals. Goodness-of-fit was assessed using R^2^ values. For predation assays, differences among *A. chinensis* developmental stages and among *T. absoluta* larval conditions were analyzed by one-way ANOVA followed by Tukey’s HSD test. Homogeneity of variance was verified using Levene’s test. For time-course mortality assays, data were fitted to a four-parameter logistic regression model [[agonist] vs. response—variable slope] to estimate median lethal times (LT_50_) with 95% confidence intervals. Differences among time-mortality curves were assessed using the extra sum-of-squares F-test. Model fit was verified by non-significant chi-square tests (*p* > 0.05).

For combination treatments evaluating predator–nematode interactions, we implemented Wu and Duncan (2022)’s method [40]. Expected mortality (*P*_E_) was calculated as *P*_E_ = *P*_0_ + (1 − *P*_0_)(*P*_1_) + (1 − *P*_0_)(1 − *P*_1_)(*P*_2_), where *P*_0_, *P*_1_, and *P*_2_ represent control, predator-alone, and nematode-alone mortality rates, respectively. The observed mortality in the combined treatment was *P*_C_. A chi-square test (χ^2^ = (*L*_O_ − *L*_E_)^2^/*L*_E_ + (*D*_O_ − *D*_E_)^2^/*D*_E_) compared observed versus expected mortality, where *L*_O_/*D*_O_ are observed dead individuals in control/combined treatments, and *L*_E_/*D*_E_ are expected values. The interaction was additive if χ^2^ < 3.84, antagonism if χ^2^ > 3.84 and *P*_C_ < *P*_E_, and synergism if χ^2^ > 3.84 and *P*_C_ > *P*_E_.

## 3. Results

### 3.1. Virulence of EPNs Against A. chinensis

**Figure 1:**
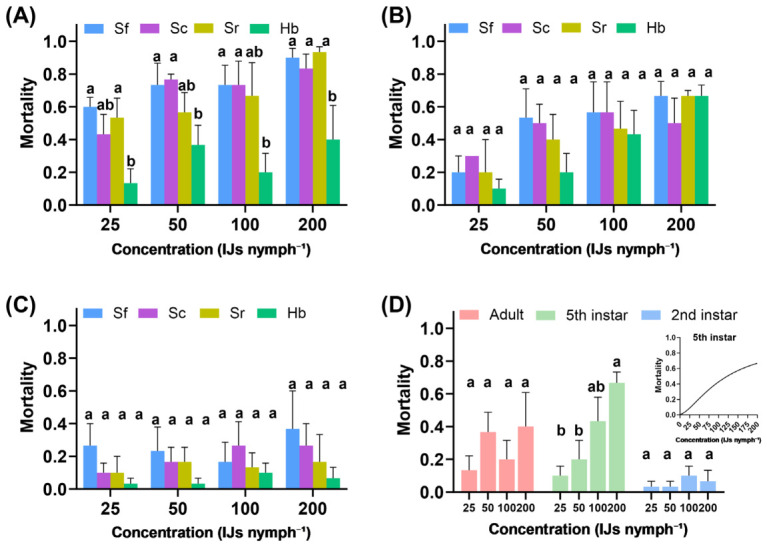
Mortality of *Arma chinensis* adults and nymphs infected by four entomopathogenic nematode species. (**A**) Adults, (**B**) 2nd-instar nymphs, and (**C**) 5th-instar nymphs exposed to *Steinernema carpocapsae* (Sc), *S. feltiae* (Sf), *S. riobrave* (Sr), and *Heterohabditis bacteriophora* (Hb) at different concentrations (25, 50, 100, 200 IJs nymph^−1^). (**D**) Concentration-dependent lethal effects of Hb across developmental stages. Data are presented as mean ± SE (*n* = 5). Different lowercase letters indicate significant differences (*p* < 0.05) among EPN species within each concentration treatment group by one-way ANOVA.

The virulence of four EPN species against *A. chinensis* varied significantly among developmental stages under confined laboratory conditions (Figure 1A–C, Table A1). For adult stink bugs, EPN species (F_3,33_ = 14.94, *p* < 0.0001) and concentration (F_11,33_ = 2.502, *p* = 0.0206) significantly affected mortality. *Heterorhabditis bacteriophora* (Hb) consistently induced the lowest mortality rates across all concentrations (*p* < 0.05; Figure 1A, Table A2), whereas the other three EPN species caused significantly higher but statistically similar mortality (*p* > 0.05). For second-instar nymphs, EPN species (F_3,33_ = 3.606, *p* = 0.0234) and concentration (F_11,33_ = 2.502, *p* = 0.0206) also significantly affected mortality (Table A1). Hb induced significantly lower mortality than *S. feltiae* (Sf; 0.058 vs. 0.258, *p* = 0.0175), but did not differ from *S. carpocapsae* (Sc; *p* = 0.1366) or *S. riobrave* (Sr; *p* = 0.5628; Table A2). For fifth-instar nymphs, only concentration significantly affected mortality (F_11,33_ = 3.712, *p* = 0.0017; Table A1).

Collectively, Hb consistently exhibited the lowest non-target virulence against all developmental stages of *A. chinensis*, and was therefore selected as the candidate species for subsequent combination trials with the predator. Further examination revealed that fifth-instar nymphs were the most susceptible to Hb, with mortality increasing in a concentration-dependent manner and reaching 67% at 200 IJs nymph^−1^ (LC_50_ = 115.8 IJs nymph^−1^; Figure 1D). In contrast, adults and second-instar nymphs exhibited relatively flat dose–responses, with mortality below 40% and 20%, respectively, and LC_50_ values > 200 IJs nymph^−1^. To minimize risk to *A. chinensis*, the lowest tested concentration of 25 IJs nymph^−1^ was selected for subsequent efficacy assays against *T. absoluta*, at which Hb induced only 5–13% mortality across predator stages.

### 3.2. Stage-Specific Predation of A. chinensis on T. absoluta

**Figure 2:**
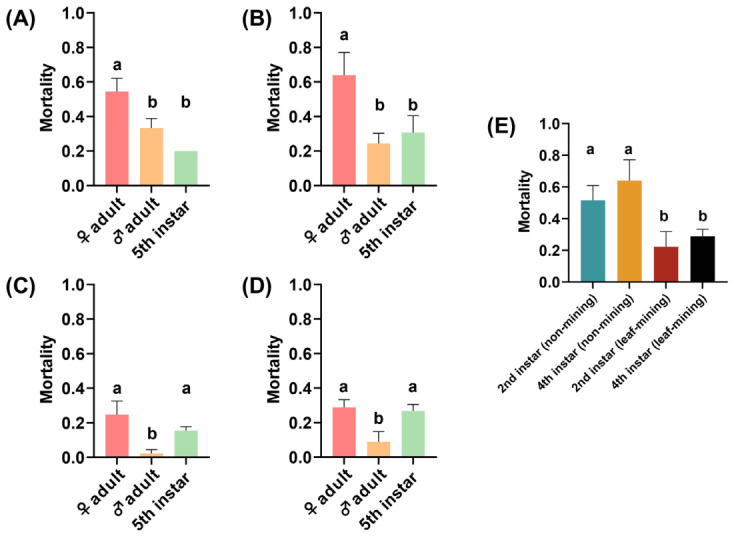
Predation efficacy of different *Arma chinensis* stages against *Tuta absoluta* larvae. Mortality of (**A**) non-mining 2nd-instar larvae, (**B**) non-mining 4th-instar larvae, (**C**) leaf-mining 2nd-instar larvae, and (**D**) leaf-mining 4th-instar larvae infected by different stages of *A. chinensis*. (**E**) Comparative efficacy of female adult *A. chinensis* against different *T. absoluta* stages. Data represent mean ± SE (*n* = 5). Different lowercase letters indicate significant differences (*p* < 0.05) by one-way ANOVA followed by LSD post hoc test. ♀ adult, female adult; ♂ adult, male adult; 5th instar, fifth-instar nymph.

*Arma chinensis* exhibited significant stage-specific predation against *T. absoluta* larvae under our assay conditions (Figure 2). When preying on second-instar larvae without leaf protection, female adults showed the highest predation rate (0.52 ± 0.09), which was significantly greater than that of male adults (0.33 ± 0.08; *p* = 0.0498) and fifth-instar nymphs (0.20 ± 0.00; *p* = 0.0036) (Tukey’s post hoc test; Figure 2A). A similar pattern occurred with unprotected fourth-instar larvae: female adults caused significantly higher mortality (0.64 ± 0.13) than male adults (0.24 ± 0.06; *p* = 0.044) and fifth-instar nymphs (0.31 ± 0.09; *p* = 0.049; Figure 2B). In treatments with leaf protection, female adults and fifth-instar nymphs induced significantly higher mortality on second-instar larvae (0.22 ± 0.09 and 0.16 ± 0.02, respectively) than male adults (0.02 ± 0.02; *p* = 0.0489 and *p* = 0.0132, respectively; Figure 2C). Similarly, against leaf-mining fourth-instar larvae, both female adults and fifth-instar nymphs showed similar efficacy (0.29 ± 0.04 and 0.27 ± 0.04, respectively) and significantly exceeded that of male adults (0.09 ± 0.06; *p* = 0.026 and *p* = 0.040, respectively; Figure 2D). Notably, although female adults maintained higher predation rates across all treatments, their efficacy declined significantly by 57–58% against leaf-mining compared to unprotected larvae (*p* < 0.05; Figure 2E).

### 3.3. Insecticidal Efficacy of EPNs Against T. absoluta

**Figure 3:**
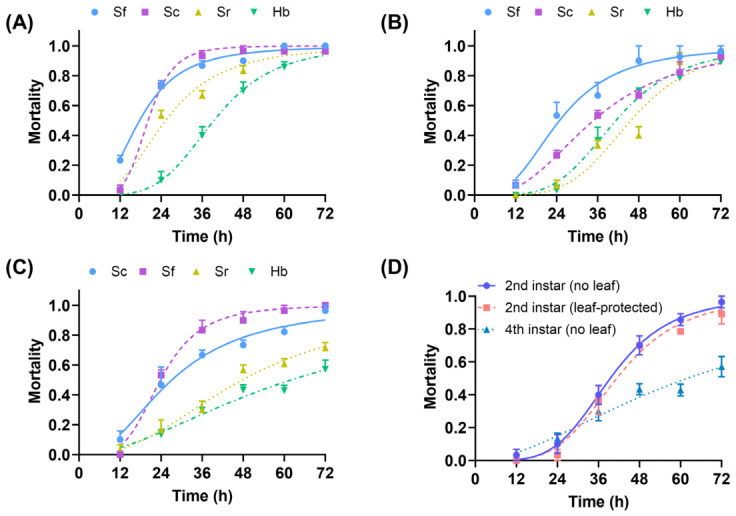
Time-course mortality of *Tuta absoluta* larvae exposed to four entomopathogenic nematode species. (**A**) Unprotected second-instar larvae, (**B**) unprotected fourth-instar larvae, and (**C**) leaf-mining second-instar larvae exposed to *Steinernema carpocapsae* (Sc), *S. feltiae* (Sf), *S. riobrave* (Sr), and *Heterorhabditis bacteriophora* (Hb). (**D**) Efficacy of Hb against different larval stages and conditions. Data are presented as mean ± SE. Curves represent four-parameter logistic regressions. Median lethal times (LT_50_) are reported in Table A3.

The four EPN species exhibited significant variation in the time course of mortality against *T. absoluta* larvae, with efficacy strongly modulated by larval instar and leaf-mining status (Figure 3 and Figure A3, Table A3). For unprotected second-instar larvae, time-course mortality increased progressively in all treatments (F_6,64_ = 60.46, *p* < 0.0001; Figure 3A). Sf achieved the fastest kill (LT_50_ = 17.7 h, 95% CI: 16.6–18.9 h), followed by Sc (LT_50_ = 20.2 h), Sr (LT_50_ = 25.3 h), and Hb (LT_50_ = 39.5 h). At 12 h, Sf induced significantly higher mortality (0.23 ± 0.03) than the other three species (*p* < 0.05; Figure A3). By 24 h, both Sc and Sf reached 73% mortality, significantly exceeding that of Hb, while Sr showed intermediate effects. At 60 h, Sc, Sf, and Sr maintained significantly higher mortality than Hb (*p* < 0.05), though by 72 h, mortality converged across all treatments (0.96–1.00, *p* > 0.05). Against unprotected fourth-instar larvae, overall susceptibility was reduced across all species (Figure 3B). Sf remained the most rapid (LT_50_ = 23.9 h, 95% CI: 22.1–25.6 h), followed by Sc (LT_50_ = 27.2 h), whereas Sr and Hb were substantially slower (47.8 h and 62.0 h, respectively) (F_6,64_ = 56.00, *p* < 0.0001). Hb mortality remained below 60% through 72 h (Figure A3).

Leaf-mining behavior further attenuated EPN efficacy (Figure 3C). Against leaf-mining second-instar larvae, LT_50_ values increased for all species, with Sf again acting most rapidly (LT_50_ = 24.9 h, 95% CI: 21.2–28.7 h), followed by Sc (34.8 h), Hb (41.4 h), and Sr (45.7 h) (F_6,64_ = 16.70, *p* < 0.0001). During the first 48 h, Sr and Hb caused negligible mortality (0–3%), significantly lower than that caused by Sc and Sf (*p* < 0.05; Figure A3). By 60 h, no significant differences in mortality rates were detected among the four species (*p* > 0.05), and by 72 h, all species achieved nearly complete mortality (0.79–1.00).

For Hb specifically, efficacy varied significantly across larval conditions (F_4,48_ = 32.20, *p* < 0.0001; Figure 3D). Unprotected and leaf-mining second-instar larvae showed similar susceptibility (LT_50_ = 39.5 h and 41.4 h, respectively), whereas fourth-instar larvae required approximately 1.5-fold longer (LT_50_ = 62.0 h). Thus, Hb exhibited clear stage- and context-dependent efficacy, with optimal performance against early instars.

### 3.4. Additive Interaction Between A. chinensis and EPNs Against T. absoluta

**Figure 4:**
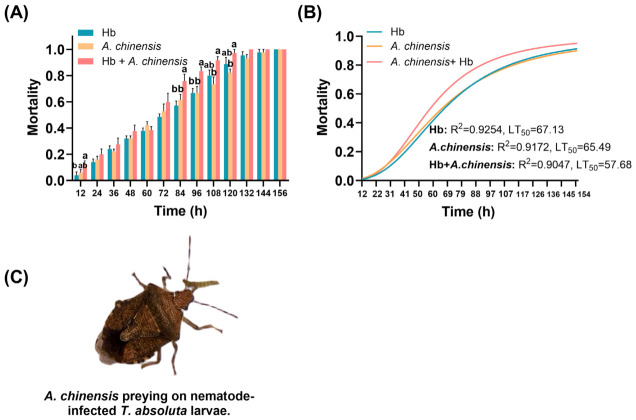
Individual and combined effects of *Arma chinensis* female adults and *Heterohabditis bacteriophora* against leaf-mining 2^nd^-instar *Tuta absoluta* larvae. (**A**) Corrected mortality rates recorded at 12 h intervals. (**B**) Time–mortality response of *T. absoluta* larvae to individual and combined applications of *H. bacteriophora* (Hb) and *A. chinensis*. (**C**) *A. chinensis* predation on Hb-infected *T. absoluta* larvae. Data represent mean ± SE (*n* = 5). Different lowercase letters indicate significant differences (*p* < 0.05) among treatments by one-way ANOVA.

**Figure A2:**
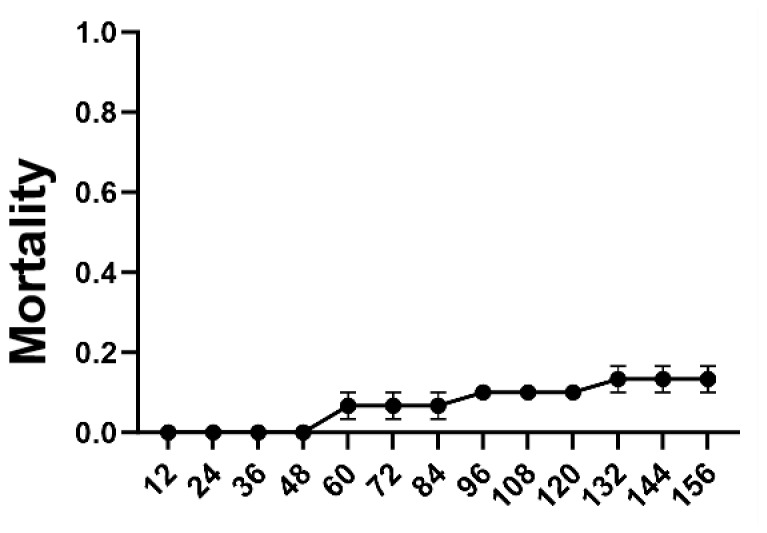
Natural mortality of *Tuta absoluta* larvae over time. Mortality rates were recorded at 12 h intervals under controlled laboratory conditions (25 ± 1 °C, 70% RH). Data represent mean ± SE (*n* = 5). No significant differences were observed among time points by one-way ANOVA (*p* > 0.05).

**Figure A3:**
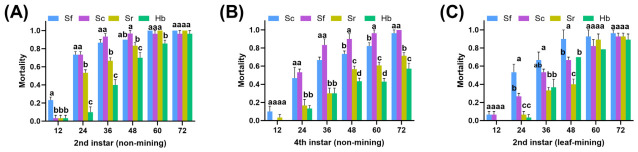
Time-dependent mortality of *Tuta absoluta* larvae induced by four entomopathogenic nematode species. (**A**) non-mining 2nd-instar larvae, (**B**) non-mining 4th-instar larvae, (**C**) leaf-mining 2nd-instar larvae exposed to *Steinernema carpocapsae* (Sc), *S. feltiae* (Sf), *S. riobrave* (Sr), and *Heterorhabditis bacteriophora* (Hb) at 25 IJs larva^−1^. Data represent mean ± SE (*n* = 5). Different lowercase letters indicate significant differences (*p* < 0.05) in mortality among EPN species at each time point by one-way ANOVA.

The combined application of *A. chinensis* and Hb exhibited a time-dependent additive effect on larval mortality of *T. absoluta* (Figure 4, Table 1). In the control group, the natural mortality of *T. absoluta* larvae remained low throughout the experiment, averaging 13% (Figure A2). At 12 h, the combined treatment caused significantly higher mortality compared to Hb alone (*p* < 0.05), though it did not differ significantly from *A. chinensis* alone (*p* > 0.05; Figure 4A). From 24 to 72 h, no significant differences were detected among treatments. Between 84 and 120 h, however, the combined treatment again showed significantly higher mortality than either agent applied alone (*p* < 0.05). All treatments eventually reached 100% mortality by 156 h. Time–mortality analysis indicated significant differences among the three treatments (F_4,176_ = 5.899, *p* = 0.0002; Figure 4B). The LT_50_ of the combined treatment was 57.68 h (95% CI: 53.04–62.18 h), which was significantly shorter than that of Hb alone (67.13 h, 95% CI: 63.30–70.90 h) or *A. chinensis* alone (65.49 h, 95% CI: 61.56–69.37 h). Interaction analysis confirmed additive effects throughout the experiment (12–156 h) rather than synergistic or antagonistic (Table 1).

Notably, one *A. chinensis* individual in the combined treatment was found dead due to nematode infection, as confirmed by dissection. In the separate observational assay, *A. chinensis* was observed preying on nematode-infected larvae in some replicates (Figure 4C), although this behavior did not alter the overall additive interaction pattern.

## 4. Discussion

Our laboratory evaluation of individual and combined biocontrol approaches highlights key operational considerations for future *T. absoluta* management strategies. The predatory bug *A. chinensis* exhibited strong efficacy against exposed (i.e., non-mining) larvae on leaf surfaces, regardless of instar, but showed limited effectiveness against larvae concealed within leaf mines. In contrast, *H. bacteriophora* achieved nearly complete control of second-instar larvae within 72 h, whether exposed or leaf-mining, but its efficacy was relatively lower against fourth-instar exposed larvae. The combined application demonstrated additive effects, producing faster overall mortality than single-agent treatments. However, two ecological interactions were noted: *H. bacteriophora* occasionally infected *A. chinensis*, and *A. chinensis* readily consumed *T. absoluta* larvae already infected by the nematodes. These findings suggest that while the combined approach accelerates pest suppression in a controlled setting, the complex interactions between these biological agents warrant careful consideration for field deployment. In practice, targeted strategies should match specific natural enemies to the most vulnerable pest stages and habitats, thereby optimizing efficacy and minimizing ecological risks.

### 4.1. Developmental Stage and Leaf-Mining Behavior Mediate Biocontrol Efficacy

Our results highlight the strong stage- and habitat-dependent differences in susceptibility of *T. absoluta* larvae to biocontrol agents. *Heterorhabditis bacteriophora* required approximately 1.5-fold longer to kill fourth-instar larvae than second-instar larvae, with LT_50_ of 62.0 h versus 39.5 h, respectively (Figure 3D; Table A3). The greater susceptibility of early instars likely reflects their thinner cuticle and weaker immune defenses, which facilitate nematode penetration and impair hemocyte-mediated defense [41]. Similar developmental resistance patterns are widely reported: early instars of *S. litura* and *S. frugiperda* are highly susceptible to *H. indica* and *S. carpocapsae*, whereas later instars or pupae are more resistant or better targeted by *S. arenarium* and *S. longicaudum* [42,43]. Pupal stages in particular often resist infection due to sclerotized integuments, as shown in *Drosophila suzukii* [44].

Leaf-mining behavior further mediates efficacy by providing shelter for larvae. Under laboratory conditions, *A. chinensis* exhibited lower predation against leaf-mining larvae (0.22–0.29) than against exposed larvae (0.52–0.64; Figure 2E). However, this limitation may be less pronounced in the field, where spatial complexity could enhance predator searching behavior. This limitation is rarely addressed in earlier studies, which typically test predation against exposed larvae. By contrast, *H. bacteriophora* readily penetrated mines and killed second-instar larvae, achieving LT_50_ values of 41.4 h for leaf-mining larvae compared to 39.5 h for exposed larvae, a modest delay that did not compromise ultimate efficacy (Figure 3C,D; Table A3). This is consistent with reports of successful EPN infections inside leaf mines [45,46,47]. Collectively, these findings illustrate the critical influence of pest stage and habitat on biocontrol efficacy, highlighting important factors to guide the design of targeted management strategies.

### 4.2. Biological Basis of Variation in Predator Performance

The differential efficacy of predators and EPNs against *T. absoluta* reflects their distinct biological traits and modes of action. Female *A. chinensis* consistently exhibited the highest predation rates across larval stages (Figure 2), a pattern reported in other predatory species such as *D. errans*, *N. pseudoferus*, and *M. basicornis*, where females consumed significantly more prey regardless of instar [48,49,50]. Similar sex-related differences are widely documented in hemipteran predators, including mirids and nabids, and are generally attributed to the elevated nutritional demands of oogenesis that promote more intensive host-searching and handling efficiency [51,52,53]. This reproductive-driven investment thus explains the consistently superior performance of females in biocontrol contexts.

### 4.3. Additive Effects in Predator–EPN Biological Control Systems

The combined use of *A. chinensis* and *H. bacteriophora* produced additive effects, reducing LT_50_ by approximately 10 h relative to either agent alone (Figure 4A,B). Such outcomes are consistent with other multi-enemy systems where additive effects occur despite intraguild predation [30,32]. This modest acceleration has limited biological significance under field conditions and should be viewed as a complementary enhancement.

However, these benefits were coupled with ecologically complex interactions (Figure 4C). *Arma chinensis* consumed nematode-infected larvae without discrimination, a form of intraguild predation that may reduce predator fitness [54]. Moreover, occasional infection of *A. chinensis* by *H. bacteriophora* was observed, indicating that top-down regulation can extend to natural enemies themselves [28].

These multi-trophic interactions resemble the mutualistic relationship between EPNs and *Fusarium solani* [29], illustrating the difficulty of extrapolating laboratory results directly to field outcomes. Instead, they establish a proof of concept and identify key interaction risks that must be managed. From an applied perspective, deployment strategies (e.g., temporal separation of releases or habitat manipulation) are needed to maximize additive effects while minimizing ecological risks, as outcomes are strongly context-dependent [27,32].

### 4.4. Study Limitations and Management Implications

This study, conducted under controlled laboratory conditions, may not fully capture the variability of field environments, where temperature, humidity, and vegetation structure could alter predator–entomopathogen interactions. Moreover, the controlled humidity (65 ± 5% RH) in our laboratory assays may overestimate EPN efficacy compared to field conditions, where humidity is often lower and more variable [24]. Specifically, the non-target virulence of EPNs against *A. chinensis* was assessed in confined arenas that prevented predator escape; therefore, the mortality rates likely represent an upper estimate of risk under forced exposure, which may be lower in a complex field habitat where predators can avoid treated zones. Moreover, we did not assess the long-term fitness effects of *A. chinensis* feeding on nematode-infected larvae, which could influence predator population dynamics in agricultural systems [54,55]. Field-based studies addressing these uncertainties remain essential.

Despite these limitations, our findings provide a framework for integrating EPNs and predatory bugs in *T. absoluta* management. Given its minimal non-target effects on *A. chinensis*, *H. bacteriophora* is a suitable nematode candidate, particularly against early instars that are highly susceptible regardless of mining status. For future foliar application, the efficacy and survival of *H. bacteriophora* will critically depend on the use of protective formulations to mitigate UV radiation and desiccation, as highlighted in recent research [22,23]. We hypothesize that introducing adult female *A. chinensis* approximately 72 h after EPN application may reduce risks of predator infection and inefficient foraging, as most larvae would have already succumbed to nematodes by this time. Complementary cultural practices, such as removing heavily infested leaves containing late-instar larvae, can further enhance overall sustainability [56].

## 5. Conclusions

This laboratory study evaluated the potential of combining the predator *A. chinensis* with EPNs for managing *T. absoluta*. Under the controlled conditions tested, *H. bacteriophora* exhibited the lowest non-target impact on *A. chinensis* among the evaluated EPN species. When applied individually, both agents showed efficacy against *T. absoluta* larvae, with their performance varying with larval stage and level of protection. Their combined use produced additive effects, reducing the LT_50_ by approximately 10 h compared with either agent alone. Importantly, interactions such as *A. chinensis* preying on EPN-infected larvae and predator mortality induced by EPN infection highlight potential ecological complexities, including intraguild predation. These findings underscore evidence of laboratory-scale compatibility between *A. chinensis* and *H. bacteriophora*, with additive effects against *T. absoluta*. For field application, however, ecological outcomes and practical efficacy will depend on factors beyond this controlled assay, such as environmental conditions, habitat complexity, and EPN formulation for foliar use. Future work should focus on field validation and refinement of application strategies to enhance compatibility and efficacy.

## Figures and Tables

**Table 1:** Time-dependent mortality and interaction effects of *Heterohabditis bacteriophora* and *Arma chinensis* against *Tuta absoluta* larvae.

Time (h)	Mortality				Interaction Analysis	
	Hb Alone	*A. chinensis* Alone	Hb + *A. chinensis* (*P*_C_)	Hb + *A. chinensis* (*P*_E_)	Effect Type	χ^2^
12	0.06	0.04	0.13	0.10	Additive effect	0.09
24	0.16	0.14	0.20	0.28	Additive effect	0.30
36	0.22	0.24	0.28	0.41	Additive effect	0.72
48	0.32	0.32	0.38	0.54	Additive effect	1.06
60	0.42	0.38	0.38	0.66	Additive effect	3.52
72	0.53	0.49	0.60	0.77	Additive effect	1.76
84	0.61	0.57	0.76	0.85	Additive effect	0.58
96	0.67	0.67	0.83	0.90	Additive effect	0.49
108	0.73	0.80	0.92	0.95	Additive effect	0.27
120	0.82	0.89	0.97	0.98	Additive effect	0.06
132	0.93	0.95	0.97	1.00	Additive effect	0.03
144	0.98	0.98	1.00	1.00	Additive effect	0.00
156	1.00	1.00	1.00	1.00	Additive effect	/

Notes: Hb, *Heterorhabditis bacteriophora*; *P*_E_, expected mortality; *P*_C_, observed mortality.

## Data Availability

The original contributions presented in this study are included in the article. Further inquiries can be directed to the corresponding authors.

## Abbreviations

The following abbreviations are used in this manuscript:

EPN	Entomopathogenic nematode
IPM	Integrated pest management
IJs	Infective juveniles
LT_50_	Median lethal time
LC_50_	Median lethal concentration
SE	Standard error
ANOVA	Analysis of variance
Sc	*Steinernema carpocapsae*
Sf	*Steinernema feltiae*
Sr	*Steinernema riobrave*
Hb	*Heterorhabditis bacteriophora*

## Appendix A

**Table A1:** Two-way ANOVA results for mortality of different developmental stages of *Arma chinensis* exposed to four entomopathogenic nematode species.

Developmental Stage	Source of Variation	df	F	*p*-Value
Adult	Concentration	11, 33	2.502	0.0206
	EPN species	3, 33	14.94	<0.0001
Second-instar nymph	Concentration	11, 33	2.502	0.0206
	EPN species	3, 33	3.606	0.0234
Fifth-instar nymph	Concentration	11, 33	3.712	0.0017
	EPN species	3, 33	1.079	0.3716

Note: df, degrees of freedom (numerator, denominator).

**Table A2:** Mean mortality of *Arma chinensis* developmental stages exposed to four entomopathogenic nematode species (pooled across concentrations).

Developmental Stage	Sc	Sf	Sr	Hb
Adult	0.692 ± 0.079 a	0.742 ± 0.079 a	0.675 ± 0.079 a	0.275 ± 0.079 b
Second-instar nymph	0.200 ± 0.064 ab	0.258 ± 0.064 a	0.142 ± 0.064 ab	0.058 ± 0.064 b
Fifth-instar nymph	0.467 ± 0.084	0.492 ± 0.084	0.433 ± 0.084	0.350 ± 0.084

Note: Data are presented as mean ± SE (pooled across concentrations of 25, 50, 100, and 200 IJs nymph^−1^). Within each developmental stage, means followed by different lowercase letters indicate significant differences among EPN species (Tukey’s HSD test, *p* < 0.05). Absence of letters indicates no significant differences among species. Sc, *Steinernema carpocapsae*; Sf, *S. feltiae*; Sr, *S. riobrave*; Hb, *Heterorhabditis bacteriophora*.

**Table A3:** Median lethal times (LT_50_) of entomopathogenic nematodes against *Tuta absoluta* larvae under different exposure conditions.

Larval Type	EPN Species	LT_50_ (h)	95% CI (h)	R^2^	F _(DFn, DFd)_	*p*-Value
2nd instar, non-mining	Sf	17.7	16.6–18.9	0.974	F_6,64_ = 60.46	<0.0001
	Sc	20.2	18.8–21.7	0.974		
	Sr	25.3	23.0–27.7	0.953		
	Hb	39.5	37.1–41.8	0.964		
4th instar, non-mining	Sf	23.9	22.1–25.6	0.965	F_6,64_ = 56.00	<0.0001
	Sc	27.2	23.4–31.0	0.891		
	Sr	47.8	44.0–52.0	0.920		
	Hb	62.0	55.7–71.6	0.877		
2nd instar, leaf-mining	Sf	24.9	21.2–28.7	0.880	F_6,64_ = 16.70	<0.0001
	Sc	34.8	32.1–37.5	0.954		
	Sr	41.4	38.8–43.9	0.957		
	Hb	45.7	41.7–49.6	0.917		
Hb only	2nd instar, non-mining	39.5	37.1–41.8	0.964	F_4,48_ = 32.20	<0.0001
	2nd instar, leaf-mining	41.4	38.8–43.9	0.957		
	4th instar, non-mining	62.0	55.7–71.6	0.877		
